# Genetic chimeras reveal the autonomy requirements for Vsx2 in embryonic retinal progenitor cells

**DOI:** 10.1186/s13064-015-0039-5

**Published:** 2015-04-27

**Authors:** Crystal L Sigulinsky, Massiell L German, Amanda M Leung, Anna M Clark, Sanghee Yun, Edward M Levine

**Affiliations:** Department of Ophthalmology and Visual Sciences, John A Moran Eye Center, University of Utah, 65 Mario Capecchi Drive, Salt Lake City, UT 84132 USA; Interdepartmental Program in Neuroscience, University of Utah, 20 North 1900 East, Salt Lake City, UT 84132 USA; Department of Neurobiology and Anatomy, University of Utah, 20 North 1900 East, Salt Lake City, UT 84132 USA

**Keywords:** Retinal development, Retinal identity, Retinal progenitor cell, Retinal ganglion cell, Vsx2, Mitf, Lhx2, *Ocular retardation*, Microphthalmia, Retina, Chimera, Progenitor, Proliferation, Neurogenesis

## Abstract

**Background:**

Vertebrate retinal development is a complex process, requiring the specification and maintenance of retinal identity, proliferative expansion of retinal progenitor cells (RPCs), and their differentiation into retinal neurons and glia. The homeobox gene *Vsx2* is expressed in RPCs and required for the proper execution of this retinal program. However, our understanding of the mechanisms by which Vsx2 does this is still rudimentary. To define the autonomy requirements for Vsx2 in the regulation of RPC properties, we generated chimeric mouse embryos comprised of wild-type and Vsx2-deficient cells.

**Results:**

We show that Vsx2 maintains retinal identity in part through the cell-autonomous repression of the retinal pigment epithelium determinant Mitf, and that Lhx2 is required cell autonomously for the ectopic Mitf expression in Vsx2-deficient cells. We also found significant cell-nonautonomous contributions to Vsx2-mediated regulation of RPC proliferation, pointing to an important role for Vsx2 in establishing a growth-promoting extracellular environment. Additionally, we report a cell-autonomous requirement for Vsx2 in controlling when neurogenesis is initiated, indicating that Vsx2 is an important mediator of neurogenic competence. Finally, the distribution of wild-type cells shifted away from RPCs and toward retinal ganglion cell precursors in patches of high Vsx2-deficient cell density to potentially compensate for the lack of fated precursors in these areas.

**Conclusions:**

Through the generation and analysis of genetic chimeras, we demonstrate that Vsx2 utilizes both cell-autonomous and cell-nonautonomous mechanisms to regulate progenitor properties in the embryonic retina. Importantly, Vsx2’s role in regulating Mitf is in part separable from its role in promoting proliferation, and proliferation is excluded as the intrinsic timer that determines when neurogenesis is initiated. These findings highlight the complexity of Vsx2 function during retinal development and provide a framework for identifying the molecular mechanisms mediating these functions.

**Electronic supplementary material:**

The online version of this article (doi:10.1186/s13064-015-0039-5) contains supplementary material, which is available to authorized users.

## Background

The vertebrate retina is one of three ocular tissues that develop from the optic vesicle, an evagination of the neuroectoderm at the level of the diencephalon. Extrinsic signals pattern the optic vesicle into three distinct domains, thereby specifying the identities of the presumptive retina, retinal pigment epithelium (RPE), and optic stalk. Growing evidence reveals that at least for the RPE and retina, initial specification alone is insufficient for proper developmental progression; rather, these identities require both active maintenance of their own gene expression programs and suppression of the other’s programs [1-6]. Further development of the retina requires coordinated proliferation and differentiation. An initially small population of specified retinal progenitor cells (RPCs) undergoes extensive proliferative expansion to generate sufficient cell numbers for the formation of a functional retina [7,8]. During this proliferative period, many of these multipotent RPCs initiate differentiation to generate retinal neurons and glia. This occurs according to an evolutionarily conserved sequence such that the six classes of retinal neurons and single glial type are each produced during limited, yet overlapping, intervals [9-13]. Disruptions in any of these processes impair proper development of the retina and visual function.

The *visual system homeobox 2* gene (*Vsx2*; *Chx10*) is an essential regulator of retinal development. Vsx2 expression demarcates the presumptive neural retina within the distal optic vesicle and is the earliest specific marker of specified RPCs [14,15]. Expression is maintained in RPCs throughout retinal development, but terminated in all postmitotic retinal cells, except bipolar cells and a subset of Müller glial cells [14,16-18]. Human patients with mutations in *Vsx2* present clinically with microphthalmia, iris colobomas, cataracts, and congenital blindness [19-26]. Mouse lines carrying spontaneous recessive mutations in the *Vsx2* gene, *ocular retardation J* (*orJ*), and the now-extinct *ocular retardation* (*or*), as well as two lines carrying missense mutations found in humans, also exhibit microphthalmia, cataracterous lenses, and coloboma and fail to form the optic nerve [27-30,4]. Vsx2 knockdown in zebrafish caused reductions in eye size and disrupted eye development [31,32]. Studies in the defined *Vsx2*-null mouse mutant, *orJ*, reveal that these defects in ocular development arise from disruptions in the execution of the retinal program, including compromised retinal identity, severely reduced RPC proliferation, delayed neurogenesis, and failure to generate bipolar cells [29,30,33,1,3].

In line with the fact that the *Vsx2* gene encodes a homeodomain, the bulk of the evidence from studies of Vsx2 activity indicates that it functions primarily as a cell-intrinsic transcription factor [19,34,35,32,36,4,37]. It remains unclear, however, which pathways or mechanisms are regulated by Vsx2 to properly execute the program of retinal development. The processes of specifying and/or maintaining retinal identity, proliferation, and neurogenesis are active simultaneously and all exert their influences upon the RPCs themselves. Thus, parsing out the mechanisms that depend on Vsx2 is challenging. These processes are also influenced by extracellular signals. It is therefore unclear whether the changes in gene expression and cell behavior in Vsx2 deficient RPCs result from changes in cell-autonomous mechanisms downstream of Vsx2, cell-nonautonomous alterations in signaling, or from both.

To address these issues, we generated mouse embryonic chimeras containing cells of wild type and Vsx2 deficient (*orJ*) origin. Genetic chimeras allow one to assess the effects of a ‘wild-type’ environment on the behavior of mutant cells as well as the effects of a ‘mutant’ environment on the behavior of wild-type cells, all in an *in vivo* context. Aggregation chimeras were previously reported for the now extinct *or* strain [38-40]. These studies revealed improved eye size and retinal structure in mutant chimeras; however, it remains unclear whether this resulted from rescued *or* cell behavior or simply compensation by wild-type cells. In the present study, we specifically assessed the behavior of *orJ* cells in chimeric retinas. We focused our analyses on the embryonic regulation of RPC properties by Vsx2: maintenance of retinal identity, RPC proliferation, and initiation of neurogenesis. We found that Vsx2 utilizes both cell-autonomous and cell-nonautonomous mechanisms in the regulation of these developmental processes.

## Results

### Production of chimeras

**Figure 1:**
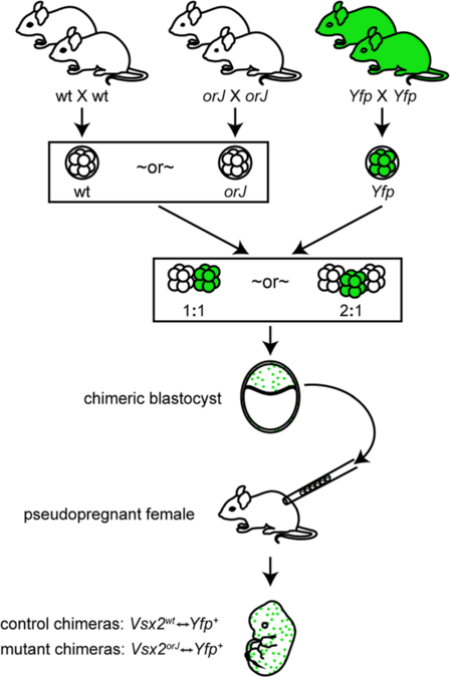
Generation of chimeras by morula aggregation. Wild-type or *orJ* morulae were aggregated overnight with morulae from the *Yfp* line at a ratio of 1:1 or 2:1. Blastocysts were injected into recipient pseudopregnant females. Embryos were harvested at select times and staged according to Theiler [103]. Abbreviations: wt, wild type.

Embryo chimeras were generated using morula aggregation techniques (Figure 1; see ‘Methods’). To distinguish between the composite cell populations, we used morulae obtained from a transgenic mouse line (yellow fluorescent protein (*Yfp*)) that ubiquitously expresses enhanced yellow fluorescent protein (EYFP) [41]. Thus, mutant chimeras were composed of EYFP-expressing wild-type cells and cells homozygous for the *orJ* allele at the *Vsx2* locus. Morulae homozygous for the wild-type allele at the *Vsx2* locus from the same background strain were used to generate control chimeras. For clarity, we refer to the EYFP-positive (EYFP+) wild-type cells as *Yfp*^+^, the EYFP-negative (EYFP−) homozygous *orJ* mutant cells as *Vsx2*^*orJ*^, and the EYFP− wild-type cells as *Vsx2*^*wt*^. Table 1 describes our efforts to generate these chimeras. For the following analyses, we analyzed 12 mutant chimeras at embryonic day 12.5 (E12.5), 1 at E14.5, and 2 at E15.5. For comparison, we also analyzed 8 control chimeras at E12.5 and 2 at E15.5.

**Table 1:** **Generation of chimeras by morula aggregation**

**Aggregation type**	**Number of morula aggregations attempted**	**Number of embryos**			
		**Implanted** ^**a**^	**Recovered**	**Evaluated for chimerism**	**Identified as chimeric** ^**b**^
1↔1 aggregations					
*Vsx2* ^*wt*^↔*Yfp* ^*+*^	58	44	8^c^	5	3
*Vsx2* ^*orJ*^↔*Yfp* ^*+*^	77	60	19^d^	13	11
2↔1 aggregations					
*Vsx2* ^*wt*^↔*Yfp* ^*+*^	120	131^e^	23^f^	21	11
*Vsx2* ^*orJ*^↔*Yfp* ^*+*^	128	128^g^	13^h^	12	10

^a^All successfully aggregated and partially aggregated (successful aggregation of 2 of the 3 morulae during 2↔1 morula aggregations) embryos were implanted into pseudopregnant females; ^b^chimeras identified by evaluation of *Yfp* ^*+*^ contribution in eye or limb sections; ^c^3 embryos exhibited light or absent eye pigmentation and were excluded from further analysis; ^d^5 embryos exhibited light or absent eye pigmentation and were excluded from further analysis; ^e^includes 102 successfully aggregated embryos, 18 partially aggregated embryos, and 11 nonaggregated embryos implanted as fillers; ^f^1 embryo was grossly underdeveloped and 1 embryo lacked eye pigmentation. Both embryos were excluded from further analysis; ^g^includes 112 successfully aggregated embryos and 16 partially aggregated embryos; ^h^1 embryo exhibited abnormal gross morphology and was excluded from further analysis.

**Figure 2:**
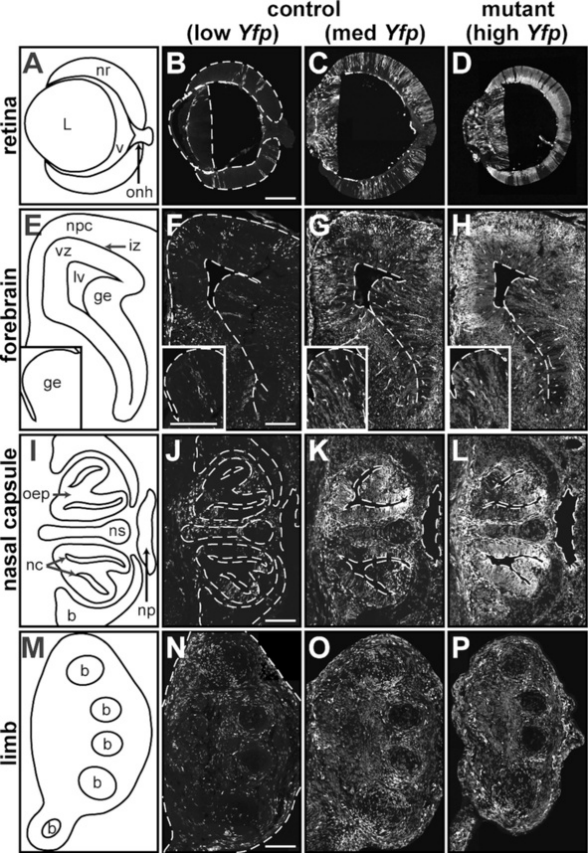
Comparison of chimerism in control and mutant chimeras across tissues. Schematic diagrams illustrating the tissue organization of the retina **(A)**, cortical epithelium **(E)**, nasal epithelium **(I)**, and limb **(M)** at E15.5. Endogenous EYFP signal in the retina **(B**-**D)**, cortical epithelium **(F**-**H)**, nasal epithelium **(J**-**L)**, and limb tissues **(N**-**P)** identifies cells contributed by the *Yfp* strain in control and mutant chimeras. Insets in E-H show enlarged region of the ganglionic eminence (inset orientation reflected along the vertical axis and rotated). Control chimera in B, F, J, and N exhibits low *Yfp* contribution in all tissues examined, while the control chimera in C, G, K, and O exhibits medium levels, and the mutant chimera in D, H, L, and P exhibits high levels of *Yfp* contribution across tissues. Dashed lines demarcate tissue boundaries. Scale bars: 200 μm. Abbreviations: b, cartilage primordium of turbinate bone **(I)** or phalangeal/metacarpal bones **(M)**; ge, ganglionic eminence (striatum); iz, intermediate zone of telencephalon; L, lens; lv, anterior horn of lateral ventricle; nc, nasal cavity; np, nasopharynx; npc, neopallial cortex; nr, neural retina; ns, cartilage primordium of nasal septum; oep; olfactory epithelium; onh, optic nerve head; v, vitreous; vz, ventricular zone of telencephalon.

The contribution and pattern of chimerism was largely consistent across tissues within individual animals (Figure 2). Both cell populations contributed to all cell compartments and in a manner that directly correlated with the magnitude of chimerism. Not unlike the previous *or* chimeras [40,38,39], only the eyes of chimeras containing *Vsx2*^*orJ*^ cells showed reductions in size (Figure 2D,H,L,P), consistent with the specificity of the *Vsx2* mutant phenotype (the smaller limb shown in Figure 2P was due to its more distal location relative to the other sections). EYFP fluorescence was more intense in neuronal layers than in progenitor layers (Figure 2D,G,H), a feature likely associated with increased or more stable EYFP expression in neurons. Importantly, *Vsx2*^*orJ*^ cells were observed in the retinas of mutant chimeras at all time points examined, suggesting that cell exclusionary mechanisms were not major influences on the patterns of chimerism. This differs from chimeric embryos containing cells mutant for the *retina and anterior neural fold homeobox* gene (*Rax*; *Rx*) in which mutant cells were excluded from the eye field, consistent with Rx having selector gene activity [42]. It also differs from chimeric embryos containing *Pax6* mutant cells, in which mutant cells were excluded from the retina at various ages in a manner consistent with mutant cell exclusion by differential cell adhesion or cell competition [43-46], and from chimeric mice containing cells heterozygous for the ribosomal protein gene *L24*, which excluded mutant cells from multiple tissues, including the retina, in a manner suggestive of cell competition [47]. The absence of mutant cell exclusion in the aggregation chimeras produced in this study afforded us the opportunity to test the autonomy characteristics of Vsx2’s requirements in regulating early retinal development.

### Vsx2-mediated regulation of retinal identity involves cell-autonomous repression of MITF

**Figure 3:**
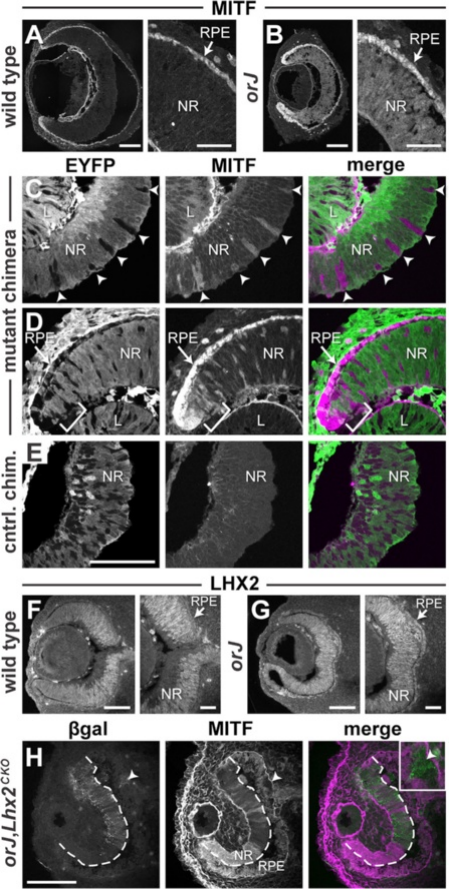
Cell-autonomous regulation of Mitf expression by Vsx2 and Lhx2. **(A**, **B)** MITF expression in E12.5 eyes of wild-type and *orJ* mice. Note the expansion of MITF expression throughout the *orJ* retina **(B)** compared to wild type **(A)**. Nonspecific staining occurs along the vitreal edges of the lens and retina, in the developing corneal epithelium and extraocular mesenchyme. See Additional file 1 for the anti-mouse immunoreactivity control. **(C**-**E)** EYFP and MITF expression in E12.5 retinas from mutant **(C, D)** and control **(E)** chimeras. MITF is detected in *Vsx2* ^*orJ*^ retinal cells throughout the retina. Expression of MITF by *Yfp* ^*+*^ cells in the retina is restricted to the extreme periphery (brackets in D), consistent with wild-type expression patterns. **(F**, **G)** LHX2 expression in wild-type and *orJ* eyes at E12.5. **(H)** β-galactosidase and MITF expression in E12.5 eyes of *orJ* mice with conditional inactivation of *Lhx2* in the retina by α-Cre. Dashed line demarcates the neural retina from the RPE. Scale bars: 100 μm (A, B, E, F, G, H); 40 μm (right panels in A, B, F, G). Abbreviations: β-gal, β-galactosidase; chim., chimera; CKO, conditional knockout; cntrl., control; EYFP, enhanced yellow fluorescent protein; L, lens; NR, neural retina; RPE, retinal pigmented epithelium.

To evaluate retinal identity in *Vsx2*^*orJ*^ cells of mutant chimeras, we examined expression of Mitf, a transcription factor required for the RPE fate and pigmentation [48,49,2]. Mitf is ectopically expressed in Vsx2-deficient RPCs and is a functional indicator of compromised retinal identity [1,3,36,4]. At E12.5, MITF expression in wild-type eyes was restricted to the developing RPE and the extreme peripheral margins of the retina (Figure 3A; Additional file 1; see Table 2 for antibody information). In contrast, MITF expression extended ectopically throughout the entire retina of E12.5 *orJ* animals (Figure 3B). Similarly, *Vsx2*^*orJ*^ cells in the retinas of E12.5 mutant chimeras displayed ectopic induction of MITF (Figure 3C,D). Neighboring *Yfp*^+^ cells (wild type) in mutant chimeras and both *Vsx2*^*wt*^ and *Yfp*^+^ cells in the retinas of control chimeras, lacked MITF expression (Figure 3C,D,E), with the exception of those located at the extreme periphery (bracketed area in Figure 3D), consistent with the pattern observed in wild-type retinas. The failure of *Vsx2*^*orJ*^ cells to downregulate MITF expression in mutant chimeras demonstrates a cell-autonomous requirement for Vsx2 in the repression of Mitf. Furthermore, these findings illustrate that compromised retinal identity persists in *Vsx2*^*orJ*^ cells of mutant chimeras.

**Table 2:** **Primary antibodies**

**Antibody**	**Target (relevant to this study)**	**Host**	**Dilution factor**	**Source**
MITF	Pigmentation marker	Mouse	400	Exalpha Biologicals (X1405M)
LHX2	RPCs	Rabbit	50	Edwin Monuki
β-gal	β-galactosidase	Rat	1,000	Nadean Brown
POU4F	RGC^a^ precursors	Goat	50	Santa Cruz (sc-6026)
ISL1	Amacrine^a^ and RGC^a^ precursors	Mouse	100	DSHB (clone 39.4D5)
OTX	Cone photoreceptor precursors	Rabbit	15,000	Chemicon (ab9566)
PTF1A	Amacrine^a^ and horizontal precursors	Guinea pig	5,000	Jane Johnson
BHLHB5	Amacrine^a^ precursors	Goat	1,000	Santa Cruz (sc-6045)
TUBB3	Neuronal^a^ precursors	Rabbit	4,000	Covance (PRB-435P)
CASP3	Apoptotic cells	Rabbit	750	BD Biosciences (clone C92-605)

^a^Labels a subset of cells in this class.

### Ectopic MITF expression in the *orJ* retina is dependent upon cell-autonomous regulation by Lhx2

The LIM homeobox gene *Lhx2* is required cell autonomously to induce or maintain expression of regional identity genes in the optic vesicle, including *Vsx2* and *Mitf* [15]. LHX2 expression was maintained in the *orJ* retina at E12.5 (Figure 3F,G), suggesting that ectopic expression of MITF in the *orJ* retina may be Lhx2 dependent. To test this possibility, we conditionally inactivated *Lhx2* in the retina of *orJ* mice using a floxed allele of *Lhx2* and the α-Cre transgene, in which Cre is driven by the retina-specific Pax6-*alpha* enhancer [50-52]. Cells that underwent recombination were identified by beta-galactosidase (βgal) expression, which originated from the recombination reporter allele, *Gt(Rosa)26Sor*^*tm1Sor*^ [53]. At E12.5, in βgal+ regions, where *Lhx2* was conditionally inactivated in the retina [54], MITF expression was absent or downregulated (Figure 3H). In contrast, βgal− regions retained ectopic MITF expression (Figure 3H), indicating that Lhx2 is required cell autonomously for ectopic expression of MITF in *orJ* retinas. *Lhx2* inactivation in a small region of the RPE (arrowhead in Figure 3H and inset) also showed loss of MITF expression, consistent with the known cell-autonomous role for Lhx2 in the induction or maintenance of MITF in the RPE [15].

### Cell-nonautonomous changes in the proliferation of *Vsx2*^*orJ*^ RPCs in mutant chimeras

**Figure 4:**
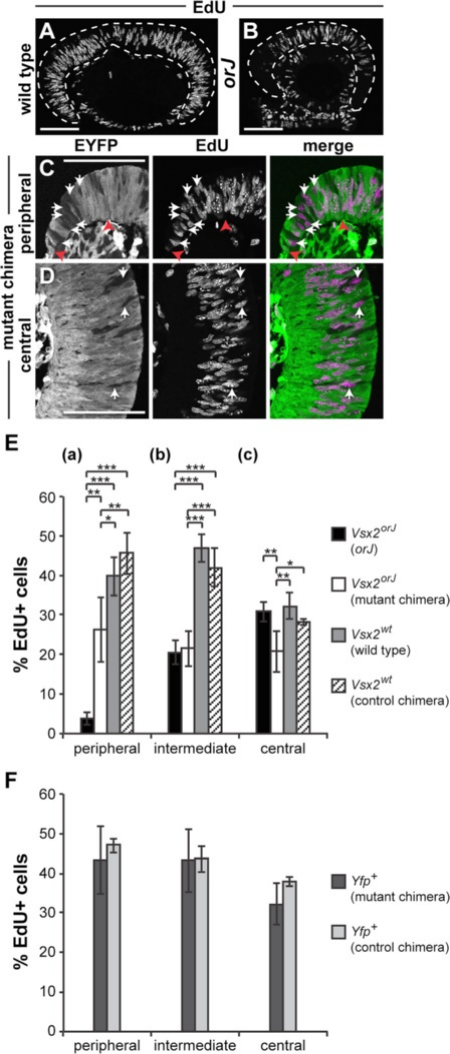
Cell-nonautonomous regulation of RPC proliferation by Vsx2. **(A**, **B)** EdU incorporation in E12.5 retinas of wild-type and *orJ* mice. Dashed lines demarcate the neural retina. **(C**, **D)** EYFP signal and EdU incorporation in retinas of mutant chimeras in peripheral **(C)** and central **(D)** regions. White arrows indicate EdU+ *Vsx2* ^*orJ*^ (EYFP−) cells in mutant chimeras. Red arrowheads in C demarcate the limits of the peripheral retinal region bounded by the adjacent corneal tissue (left arrowhead) and the intermediate retinal region (right arrowhead). **(E)** Quantification of EdU+ *Vsx2* ^*orJ*^ and *Vsx2* ^*wt*^ cells at E12.5 in peripheral ***(a)***, intermediate ***(b)***, and central ***(c)*** regions of retinas from *orJ*, wild type, and chimeras. *P* values calculated by Tukey-Kramer HSD comparison of pairs within, but not across, regions. **(F)** Quantification of EdU+ *Yfp* ^*+*^ cells at E12.5 in peripheral, intermediate, and central regions of retinas from mutant and control chimeras. *P* values calculated using Student’s unpaired *t*-test or Welch’s two-sample *t*-test, as appropriate (based on results of an F-test of variances) within, but not across, regions. Bars represent mean ± standard deviation. *P < 0.04, **P < 0.006, ***P < 0.0001. Nonsignificant changes (all *P* > 0.2) are not shown. Scale bars: 100 μm. Abbreviations: EYFP, enhanced yellow fluorescent protein.

Previous *or* mutant chimeras exhibited increased eye size compared to germline *or* mutants, but eye size of chimeras still varied inversely with *or* cell contribution [38-40]. Thus, to determine whether the proliferation of *Vsx2*^*orJ*^ RPCs was rescued in our mutant chimeras, we assayed for S-phase incorporation of the thymidine analog, EdU, following a brief labeling pulse at E12.5. In wild-type retinas, EdU incorporation was robust throughout the retina (Figure 4A). We observed robust labeling in central regions of *orJ* retinas, but little to no labeling in the periphery (Figure 4B), consistent with previous reports [30,29,55,56]. In contrast, *Vsx2*^*orJ*^ cells were labeled with EdU in both central and peripheral regions of mutant chimeras (Figure 4C,D).

To examine the proliferation of *Vsx2*^*orJ*^ cells further, we quantified the proportion of EdU+ cells in both *Vsx2*^*orJ*^ and *Vsx2*^*wt*^ populations across different regions (peripheral, intermediate, central) in chimeric, *orJ*, and wild-type retinas (Figure 4E; Table 3, see ‘Methods’ for details). In the peripheral region, we observed a dramatic sixfold increase in the EdU labeling index of *Vsx2*^*orJ*^ cells in mutant chimeras compared to *orJ* (Figure 4E(*a*)), consistent with a robust rescue of *Vsx2*^*orJ*^ proliferation. This rescue failed to reach wild-type levels as indicated by the EdU labeling index of *Vsx2*^*wt*^ cells in control chimeras (Figure 4E(*a*)). This was not due to a suboptimal environment provided by the *Yfp*^+^ cells since the EdU labeling indices were not different for *Vsx2*^*wt*^ cells in control chimera and wild-type retinas (Figure 4E(*a*)).

**Table 3:** **EdU labeling analysis at E12.5**

**Cell type**	**Environment**	**Number of animals**	**Peripheral**	**Intermediate**	**Central**
			**% EdU +** ^**a**^	**% EdU +** ^**a**^	**% EdU +** ^**a**^
			**Number of cells**	**Number of cells**	**Number of cells**
*Vsx2* ^*orJ*^	*orJ*	4	4% ± 2%	20% ± 3%	31% ± 2%
			1,880 cells	2,554 cells	2,490 cells
*Vsx2* ^*orJ*^	Mutant chimera	5	26% ± 8%	21% ± 4%	21% ± 5%
			732 cells	798 cells	519 cells
*Vsx2* ^*wt*^	Wild type	3	40% ± 5%	47% ± 4%	32% ± 3%
			1,390 cells	2,207 cells	2,186 cells
*Vsx2* ^*wt*^	Control chimera	4	46% ± 5%	42% ± 5%	28% ± 1%
			594 cells	1,093 cells	883 cells
*Yfp* ^*+*^	Mutant chimera	3	43% ± 8%	43% ± 8%	32% ± 5%
			1,209 cells	2,608 cells	2,169 cells
*Yfp* ^*+*^	Control chimera	4	47% ± 2%	44% ± 3%	38% ± 1%
			809 cells	1,048 cells	1,378 cells

^a^%EdU+ provided as mean ± standard deviation.

Unexpectedly, we observed a 30% reduction in EdU+ *Vsx2*^*orJ*^ cells in the central region of mutant chimeras compared to the same region in *orJ* retinas (Figure 4E(*c*)). This was not observed for *Vsx2*^*wt*^ cells; the percentage of EdU+ cells was similar between control chimera and wild-type retinas (Figure 4E(*c*)). These findings reveal a novel, cell-nonautonomous inhibition of *Vsx2*^*orJ*^ proliferation in mutant chimeras.

In contrast to the peripheral and central regions, *Vsx2*^*orJ*^ cells in the intermediate region exhibited similar EdU labeling indices in mutant chimeras and *orJ* (Figure 4E(*b*)). Notably, these values are well below that of *Vsx2*^*wt*^ cells (Figure 4E(*b*)). As with the central and peripheral regions, the environment provided by the *Yfp*^+^ cells did not have a significant effect on the *Vsx2*^*wt*^ cells (Figure 4E(*b*)). The general lack of environmental influences on proliferation in the intermediate region suggest that the proliferation deficiency of *Vsx2*^*orJ*^ cells in this region was largely due to cell-autonomous changes. Finally, the environment provided by the *Vsx2*^*orJ*^ cells did not have an effect on the proliferation of wild-type cells since the EdU labeling indices of *Yfp*^+^ cells in mutant and control chimeras were similar in each region (Figure 4F).

### Vsx2 cell autonomously promotes initiation of neurogenesis

Retinal neurogenesis in the mouse initiates at approximately E11 in the central retina, dorsal to the optic stalk, and continues in a peripherally spreading wave [57]. By E12.5, neurogenesis is active throughout the central retina of wild-type mice but has yet to initiate in *orJ* retinas [58,28,4,30]. This provides us with a window to evaluate the ability of a wild-type environment to restore neurogenesis in *Vsx2*^*orJ*^ cells.

**Figure 5:**
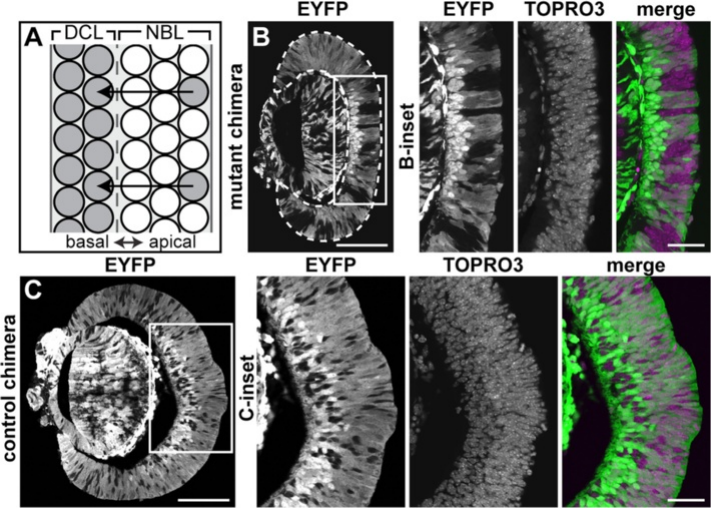
*Vsx2* ^*orJ*^ cells are rarely located in the differentiated cell layer of chimeras at E12.5. **(A)** Diagram illustrating the relationship between differentiation status and apical-basal position within the retina. Nascent postmitotic cells (sparse gray cells at apical surface) migrate basally to establish a distinct differentiated cell layer composed of postmitotic neurons. **(B**, **C)** Distribution of EYFP+ and EYFP− cells in E12.5 retinas of mutant **(B)** and control **(C)** chimeras. (B-inset, C-inset) Boxed areas in B and C. The nuclear marker TOPRO3 demonstrates that the EYFP− regions are not holes, but *Vsx2* ^*orJ*^ (B-inset) or *Vsx2* ^*wt*^ (C-inset) cells. Scale bars: 100 μm **(B, C)**; 40 μm (insets). Abbreviations: DCL, differentiated cell layer; EYFP, enhanced yellow fluorescent protein; NBL, neuroblast layer.

We first compared the apical-basal location of *Yfp*^+^ and *Vsx2*^*orJ*^ cells in the retinas of mutant chimeras, as this reflects a cell’s differentiation status during the neurogenic period. Behind the neurogenic wave front, nascent postmitotic cells migrate basally to establish a distinct differentiated cell layer, leaving progenitors in an overlying apical neuroblast layer (Figure 5A). In the preneurogenic (peripheral) retina of E12.5 mutant chimeras, *Vsx2*^*orJ*^ cells occupied various positions along the apical-basal axis (Figure 5B). Within the neurogenic (central) region of mutant chimeras, *Yfp*^+^ cells dominated the basal differentiated cell layer, while *Vsx2*^*orJ*^ cells appeared to be restricted apically, within the neuroblast layer (Figure 5B, B-inset). In contrast, *Vsx2*^*wt*^ cells in control chimeras readily populated the differentiated cell layer, in addition to the neuroblast layer (Figure 5C, C-inset). These findings suggest that in mutant chimeras, *Vsx2*^*orJ*^ retinal cells have yet to initiate neurogenesis.

**Figure 6:**
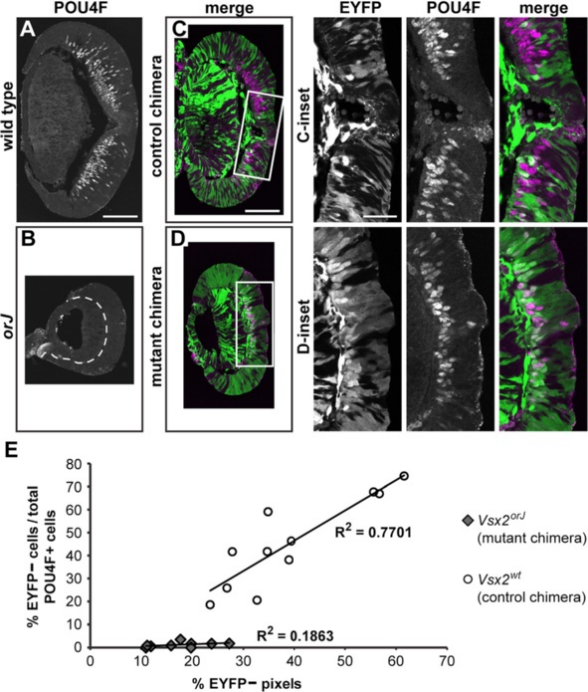
*Vsx2* ^*orJ*^ RPCs do not produce RGC precursors in mutant chimeras at E12.5. **(A**-**D)** Expression of POU4F in retinas of wild type **(A)**, *orJ* **(B)**, control chimeras **(C)**, and mutant chimeras **(D)** at E12.5. (C-inset, D-inset) Boxed areas in C and D. Dashed lines in B delineate the retina from adjacent lens tissue. Images are maximum Z-projections of confocal scans. **(E)** Bivariate graph showing the relationships between the percentages of EYFP− cells contributing to the RGC population (y-axis) and the percentages of EYFP− pixels contributing to the apical-basal extent of retinal tissue (x-axis) in the regions where POU4F+ cells were counted in mutant and control chimeras. Pixels serve as a proxy for relative contribution of EYFP− cells to the retina. Each data point was collected from a single retinal section (mutant chimeras: *n* = 9, 5 retinas; control chimeras: *n* = 11, 4 retinas) Scale bars: 100 μm **(A, C)**; 40 μm (insets). Abbreviations: EYFP, enhanced yellow fluorescent protein.

It is possible, however, that *Vsx2*^*orJ*^ cells differentiated but failed to localize to the differentiated cell layer. To address this possibility, we first examined the differentiation of retinal ganglion cells (RGCs), the earliest born cell class in the retina. Staining for POU4F, which labels the majority of newly differentiated RGC precursors [59-63], revealed that RGCs were abundant in the central regions of wild-type retinas by E12.5 (Figure 6A). However, POU4F+ RGCs were absent in *orJ* retinas at this age, clearly revealing the delayed initiation of neurogenesis (Figure 6B). In mutant chimeras, many POU4F+ RGCs were present in the central retina, but we rarely observed *Vsx2*^*orJ*^ cells contributing to this population (Figure 6D, D-inset). This is in stark contrast to control chimeras, where the POU4F+ RGC population was composed of *Yfp*^+^ and *Vsx2*^*wt*^ cells (Figure 6C, C-inset). We quantified these differences by calculating the percentage of *Vsx2*^*orJ*^ and *Vsx2*^*wt*^ cells that comprised the POU4F+ RGC population (Figure 6E; see ‘Methods’). Whereas *Vsx2*^*wt*^ cells produced POU4F+ cells in a manner consistent with their overall contribution to the retina in the same region (Figure 6E; *R*^2^ = 0.7701), *Vsx2*^*orJ*^ cells produced few, if any, POU4F+ cells, regardless of their contribution to the retina at this stage of development (Figure 6E; *R*^2^ = 0.1863), as indicated by the significantly different slopes of the least squares fit regression lines (*P* = 0.02). While the graph shows that *Vsx2*^*orJ*^ cells tended to contribute less to the retina than the *Vsx2*^*wt*^ cells, we cannot definitively conclude this because a statistical comparison of chimerism in other tissues was not done. However, this apparent observation is not surprising since *Vsx2*^*orJ*^ proliferation was significantly lower in the same regions (*central*, *intermediate*) of the mutant chimeras at this age (Figure 4E(*b*,*c*)).

**Figure 7:**
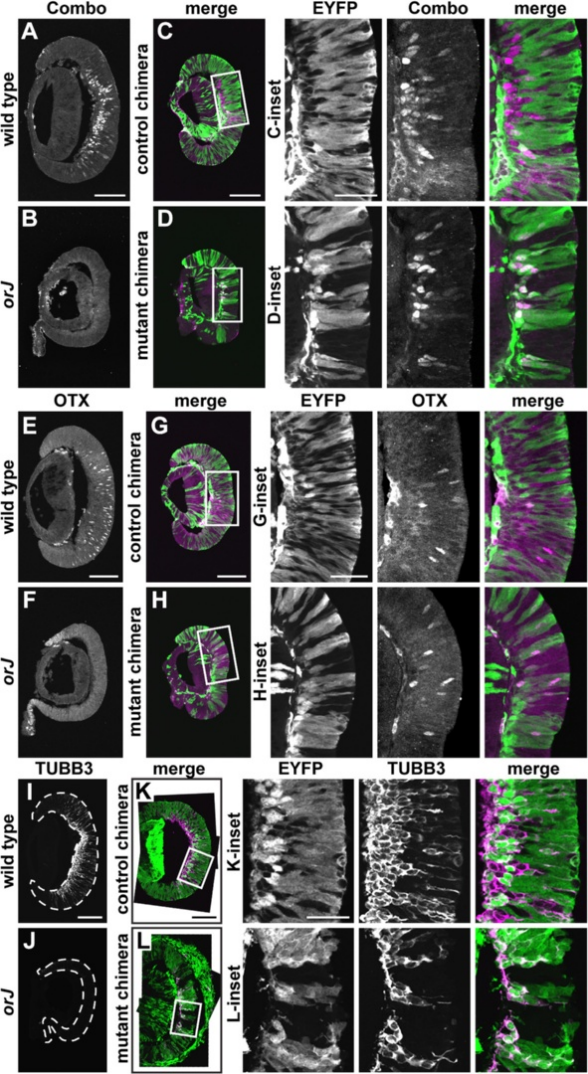
*Vsx2* ^*orJ*^ cells fail to express markers of postmitotic precursors at E12.5. Expression of the combination marker stain **(A**-**D)**, OTX **(E**-**H)**, and TUBB3 **(I-L)** in retinas of wild type (A, E, I), orJ (B, F, J), control chimeras (C, G, K), and mutant chimeras (D, H, L) at E12.5. (Insets) Boxed areas in C, D, G, H, K, and L. Combo stain represents simultaneous staining for ISL1, POU4F, PTF1A, and BHLHB5. The OTX antibody recognizes both OTX1 and OTX2, but the strong, scattered OTX2 expression in postmitotic precursors is readily distinguished from the more peripheral OTX1 upregulation that occurs in the *orJ* retina **(E, F)**. Dashed lines in I and J delineate the boundary of retinal tissue. All images are maximum Z-projections of confocal scans. Scale bars: 100 μm (A, C, E, G, I, K); 40 μm (insets). Abbreviations: EYFP, enhanced yellow fluorescent protein.

To rule out the possibility that *Vsx2*^*orJ*^ cells differentiate in the mutant chimera, but skip the RGC fate, we also surveyed the differentiation of the other early born retinal cell classes using a panel of precursor markers: ISL1 to label subsets of RGC and amacrine precursors [64,65], PTF1A to label subsets of amacrine and horizontal precursors [66-68], BHLHB5 to label subsets of amacrine precursors [69], and pan-OTX to label cone photoreceptor precursors [70-72] (Figure 7; Table 2). To limit the possibility of missing differentiated cells due to low levels of neurogenesis in the *Vsx2*^*orJ*^ cell population, we stained for these precursor markers and POU4F simultaneously (Figure 7A,B,C,D). OTX was stained separately because of species incompatibility with the other antibodies (Figure 7E,F,G,H). Although wild-type retinas and control chimeras exhibited differentiation of these early born cell types at E12.5 (Figure 7A,C,E,G), *orJ* retinas did not (Figure 7B,F). Critically, *Vsx2*^*orJ*^ cells in E12.5 mutant chimeras also lacked expression of these markers (Figure 7D,H). As a final test, we examined the expression of class III β-tubulin (TUBB3), a general marker of postmitotic neurons [73,74] that reliably reflects the progression of retinal neurogenesis [57,58,75], and is expressed in the wild-type retina and *Vsx2*^*wt*^ and *Yfp*^+^ cells in the control chimera (Figure 7I,K). Consistent with the other markers, TUBB3 expression was absent in the *orJ* retina and in *Vsx2*^*orJ*^ cells in the mutant chimera (Figure 7J,L). Together, these findings demonstrate that *Vsx2*^*orJ*^ cells fail to participate in retinal neurogenesis at this stage of development, even in the presence of neighboring wild-type cells undergoing neurogenesis.

**Figure 8:**
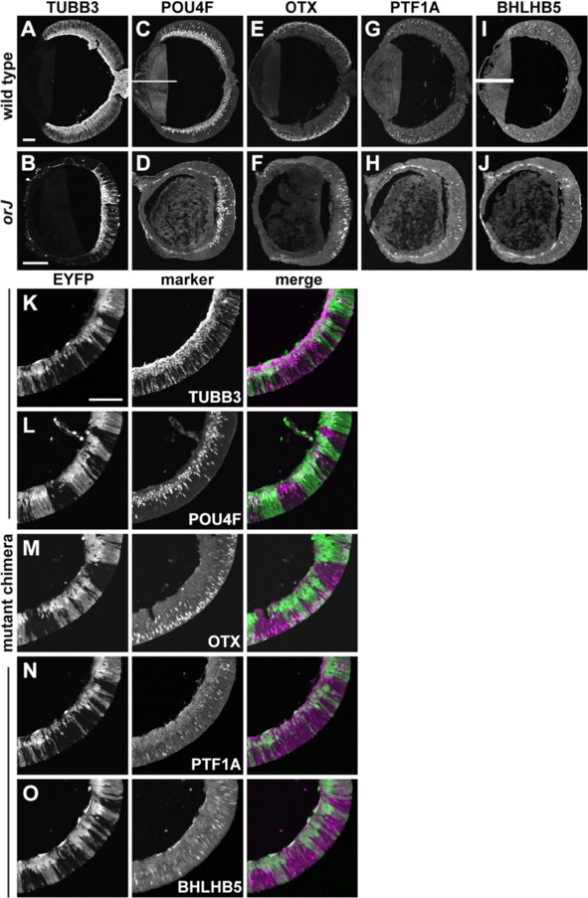
*Vsx2* ^*orJ*^ cells contribute to all early born retinal cell types in mutant chimeras at E15.5. **(A**-**J)** Expression of TUBB3 **(A, B)**, POU4F **(C, D)**, OTX **(E, F)**, PTF1A **(G, H)**, and BHLHB5 **(I, J)** in wild-type **(A, C, E, G, I)** and *orJ* **(B, D, F, H, J)** retinas. White bars in C and I are the consequence of nonoverlapping fields of view during image capture. **(K**-**O)** Expression of TUBB3 **(K)**, POU4F **(L)**, OTX **(M)**, PTF1A **(N)** and BHLHB5 **(O)** in retinas of mutant chimeras at E15.5. All markers are detected in *Yfp* ^*+*^ and *Vsx2* ^*orJ*^ cells. Scale bars: 100 μm. Abbreviations: EYFP, enhanced yellow fluorescent protein.

Because neurogenesis eventually gets underway in the *orJ* retina [29,30,33,58,76], we predicted that *Vsx2*^*orJ*^ cells would also differentiate in chimeras. In the *orJ* retina, all of the early born retinal cell classes are detectable by E15.5 (Figure 8A,B,C,D,E,F,G,H,I,J). As predicted, many *Vsx2*^*orJ*^ cells expressed TUBB3 and contributed to the expanding differentiated cell layer in the E15.5 mutant chimeras (Figure 8K). Furthermore, *Vsx2*^*orJ*^ cells in mutant chimeras contributed to all of the early born retinal cell types at E15.5 (Figure 8L,M,N,O). Neurogenesis of *Vsx2*^*wt*^ cells in control chimeras are provided for comparison (Additional file 2).

**Figure 9:**
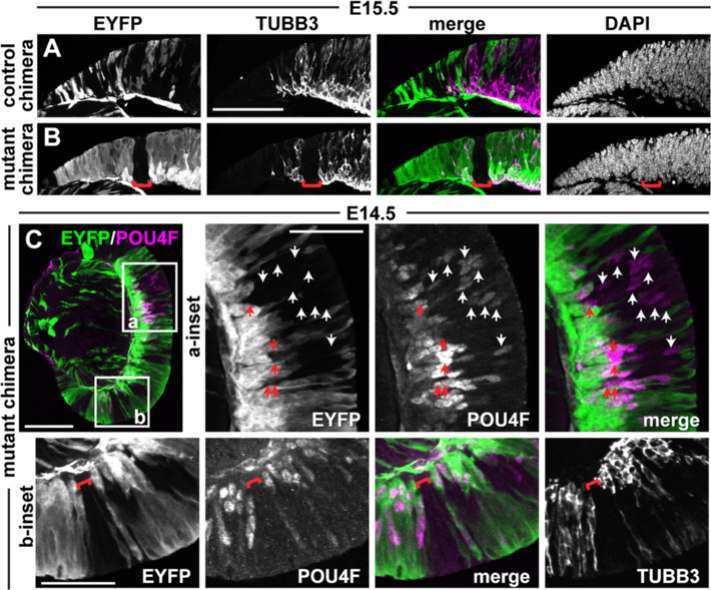
Delayed neurogenesis of *Vsx2* ^*orJ*^ cells persists in the periphery of mutant chimeras. **(A**, **B)** EYFP and TUBB3 expression in the peripheral retina of control **(A)** and mutant **(B)** chimeras at E15.5. DAPI staining reveals a patch of *Vsx2* ^*orJ*^ cells lacking TUBB3 (red brackets in B). **(C)** EYFP (green) and POU4F (magenta) expression in retina of a mutant chimera at E14.5. (a-inset, b-inset) Boxed regions in C. Arrows in a-inset indicate POU4F+ *Vsx2* ^*orJ*^ cells in the neuroblast (white arrows) or differentiated (red arrows) cell layers. Red brackets in b-inset indicate peripheral patch of *Vsx2* ^*orJ*^ cells lacking POU4F and TUBB3. Scale bars: 100 μm. Abbreviations: EYFP, enhanced yellow fluorescent protein.

Despite active neurogenesis in *orJ* retinas at E15.5, the extent of differentiation remained more centrally restricted than in wild-type retinas (Figure 8A,B,C,D,E,F,G,H,I,J), consistent with delayed initiation of the central-to-peripheral wave of neurogenesis [58]. Similarly, in retinas of E15.5 mutant chimeras, peripheral patches of *Vsx2*^*orJ*^ cells coincided with gaps in the neuronal marker TUBB3, revealing their delayed differentiation relative to peripheral *Yfp*^+^ and central *Vsx2*^*orJ*^ cells (Figure 9A,B). Cell-type-specific markers also showed a similar trend, but it was less obvious due to the sparse nature of their patterns at the leading edge and random positioning of *Vsx2*^*orJ*^ cells in chimeric retinas (data not shown). To confirm the lag in differentiation of peripheral *Vsx2*^*orJ*^ cells relative to adjacent *Yfp*^+^ cells, we examined an earlier age that exhibits a more pronounced difference in the peripheral extent of neurogenesis. In an E14.5 mutant chimera, a number of *Vsx2*^*orJ*^ cells located within the central retina were POU4F+, indicating differentiation as RGCs (Figure 9C, a-inset). In contrast, patches of *Vsx2*^*orJ*^ cells in an intermediate region lacked POU4F and TUBB3 expression, despite expression of both markers in more peripheral *Yfp*^+^ cells (red bracket, Figure 9C, b-inset). Interestingly, only a few POU4F+ *Vsx2*^*orJ*^ cells were detected in the differentiated cell layer of the central retina (red arrows, Figure 9C, a-inset); most were still localized to the neuroblast layer (white arrows, Figure 9C, a-inset), consistent with a more recent birthdate. Together, these analyses reveal a cell-autonomous delay in neurogenesis of *Vsx2*^*orJ*^ cells in retinas of mutant chimeras that is consistent with the delayed progression of neurogenesis in the *orJ* retina.

### Cell-nonautonomous effects on the neurogenic output of wild-type cells

**Figure 10:**
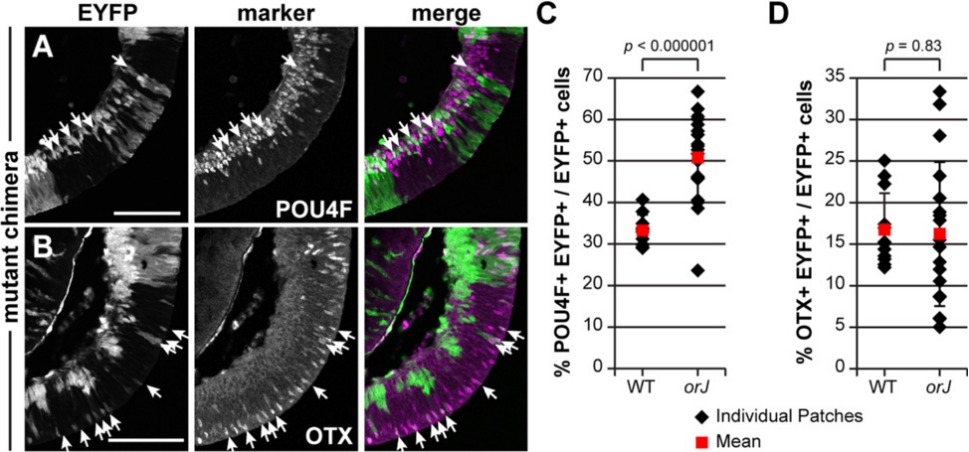
Altered cell type distribution of *Yfp* ^*+*^ cells in regions of high *Vsx2* ^*orJ*^ contribution in chimeras. Expression of POU4F **(A)** and OTX **(B)** in retinas of mutant chimeras at E15.5. Quantification of POU4F+ **(C)** and OTX+ **(D)** *Yfp* ^*+*^ cells as a function of patch type in retinas of mutant chimeras at E15.5. Black diamonds represent individual patch values and illustrate the variation within patches of the same type. Red squares represent mean ± standard deviation. *P* values calculated by Student’s unpaired *t*-test or Welch’s two-sample *t*-test, as appropriate (based on results of an F-test of variances). Scale bars: 100 μm. Abbreviations: EYFP, enhanced yellow fluorescent protein.

Our examination of E15.5 mutant chimeras revealed an unexpected change in the distribution of wild-type cells when exposed to large patches of *Vsx2*^*orJ*^ cells (Figure 10). In these areas, few *Yfp*^+^ cells were observed in the neuroblast layer (progenitor zone) of mutant chimeras. Rather, *Yfp*^+^ cells were predominantly found in the differentiated cell layer or scattered near the apical edge of the neuroblast layer and expressed POU4F or OTX (white arrows, Figure 10A,B). Only in areas of low *Vsx2*^*orJ*^ contribution were patches of *Yfp*^+^ cells found to span the entire apical-basal width of the retina and populate the neuroblast layer (Figure 10A,B). These findings suggest that a cell-nonautonomous process influenced the differentiation or survival of wild-type RPCs when exposed to an *orJ* environment.

Cell death is unlikely to account for the absence of wild-type RPCs in the *orJ* patches because activated caspase-3-positive (CASP3+) cells were rarely detected in the retinas of mutant chimeras at E12.5, E14.5, and E15.5 (Additional file 3). CASP3 staining was observed in the peripheral retina of an E12.5 mutant chimera, but this was confined to *Vsx2*^*orJ*^ cells (Additional file 3F). Similarly, CASP3+ cells were observed in E12.5 *orJ* retinas (Additional file 3B,C), consistent with previous reports of a delayed burst of cell death in the *orJ* retina [28].

We suspected that regions of high *Vsx2*^*orJ*^ cell density promoted a shift toward precursor production in the enclosed wild-type (*Yfp*^+^) progenitors. To address this, we calculated the percentages of *Yfp*^*+*^ cells that were POU4F+ or OTX+ in the mutant chimeras at E15.5 and segregated the data into regions that were predominantly composed of *Yfp*^+^ cells (wild-type environments) or *Vsx2*^*orJ*^ cells (*orJ* environments; see ‘Methods’). We did not quantify PTF1A+ and BHLHB5+ cell populations due to their smaller size and reduced probability of finding *Yfp*^+^ cells to evaluate. In wild-type environments with little *orJ* influence, approximately a third of the *Yfp*^*+*^ cells were POU4F+, whereas half were POU4F+ in *orJ* environments (Figure 10C; Table 4). Despite this overrepresentation, overproduction of RGC precursors at the tissue level was not apparent at this age (Figure 10A) or earlier (Figure 6C,D). Interestingly, the overrepresentation of RGC precursors in the *Yfp*^+^ population was specific; the proportion of *Yfp*^+^ OTX+ precursors was unchanged between the two environments, although individual *orJ* environments exhibited greater variation (Figure 10B,D; Table 4). These observations are consistent with a model in which the *orJ* environment enhanced the early neurogenic output of the enclosed wild-type cells at the expense of their maintenance as RPCs. This effect is likely to have manifested at or after the normal onset of neurogenesis since the EdU labeling indices of *Yfp*^+^ cells in mutant and control chimeras were not different at E12.5 (Figure 4F).

**Table 4:** **Neurogenesis analysis at E15.5**

**Cell type**	**Environment**	**Number of animals**	**Number of patches**	**Number of cells**	**% Marker +** ^a^
POU4F					
*Yfp* ^*+*^	Wild type	1	10	1,635	33% ± 4%
*Yfp* ^*+*^	*orJ*	2	19	1,022	51% ± 10%
OTX					
*Yfp* ^*+*^	Wild type	2	12	1,485	17% ± 4%
*Yfp* ^*+*^	*orJ*	2	18	1,012	16% ± 9%

^a^% Marker + provided as mean ± standard deviation.

## Discussion

In this study, we show that chimera analysis provides a powerful, unbiased approach to determine the extent of extrinsic influence Vsx2 exerts in its regulation of RPC properties. We determined the autonomy of Vsx2 function during the embryonic stages of retinal development, an important step in placing Vsx2 in the context of known regulatory pathways driving the maintenance of retinal identity, RPC proliferation, and initiation of neurogenesis.

### Vsx2 is part of a cell-autonomous mini-circuit controlling retinal identity

A primary role for Vsx2 in the maintenance of retinal identity is to restrict expression of non-retinal gene expression programs, in large part through its regulation of Mitf activity [1,3,4]. Genetic removal of *Mitf* in *orJ* retinas improves retinal development, while genetically increasing *Mitf* gene dosage in *orJ* retinas further exacerbates the pigmentation program, demonstrating that aberrant Mitf expression is a major contributor to the *orJ* phenotype [1,77,36,4]. Mitf expression in the eye is also regulated by extrinsic signals, including FGF, Wnt-β-catenin, and the TGFβ family member, Activin [2,78-82]. It therefore remained possible that Vsx2 influences extrinsic signals to repress Mitf. In the present study, we show that *Vsx2*^*orJ*^ cells fail to downregulate MITF expression in mutant chimeras, emphasizing the critical cell-autonomous role for Vsx2 in mediating this response. This cell-autonomous repression of MITF by Vsx2 is consistent with reports that *Mitf* is a direct transcriptional target of Vsx2 [36,4].

FGF signaling is required for Vsx2 expression in the presumptive retina [2,83], and FGFs are also sufficient to repress Mitf expression and promote the transdifferentiation of embryonic RPE into retina [2,1,84,6]. In the *orJ* retina, expression of *Vsx2* transcript is maintained [58,76], which suggests that FGF signaling is still active. The failure of FGFs to repress Mitf and restore the retinal program in *orJ* retinal explants reveals that Vsx2 mediates much of these functions [1].

**Figure 11:**
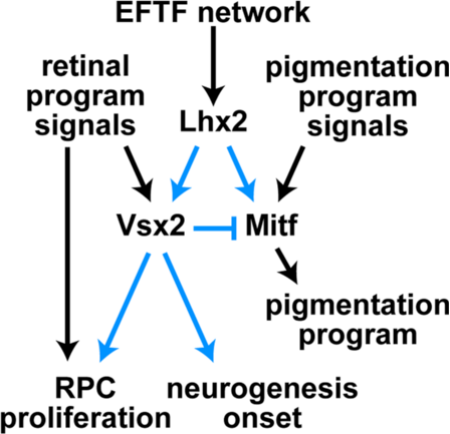
Model of Vsx2 function during early retinal development. As a part of the eye field transcription factor (EFTF) network, Lhx2 provides the competence for the optic neuroepithelium to express Mitf and Vsx2. Pigmentation program signals (that is, TGF-beta family) activate Mitf, and in turn, retinal program signals (FGFs, BMPs) activate Vsx2. Vsx2 then represses Mitf in the retinal domain. Once the retinal program is initiated, Vsx2 promotes RPC proliferation through cell-autonomous and cell-nonautonomous mechanisms, and the timing of onset of neurogenesis through a cell-autonomous mechanism. Finally, the timing mechanism for neurogenesis onset does not appear to be dependent on the mechanisms regulated by Vsx2 to promote proliferation. Abbreviations: RPC, retinal progenitor cells.

While the absence of VSX2 protein in *orJ* cells explains the failure to downregulate MITF, it is not clear why MITF expression persists. In other words, what underlies the competence of RPCs to permit such expression? In the present study, we found that Lhx2 is required for Mitf expression in *orJ* RPCs. Because Lhx2 is also required cell autonomously for Vsx2 expression in the retina and for Mitf in the RPE [15], we propose a model in which Lhx2 is necessary for the expression of both Vsx2 and Mitf in the retina (Figure 11). During eye field patterning, the combination of Lhx2 expression in the optic neuroepithelium and RPE-promoting signals from the surrounding mesenchyme promote Mitf expression throughout the optic vesicle. Upon contact with the surface ectoderm, FGF signaling promotes Vsx2 in the presumptive retina, which in turn represses Mitf. A reciprocal inhibitory regulation/repression does not appear to be present, at least in the context of the retina, as *Vsx2* mRNA expression is maintained in the *orJ* retina, despite persistent Mitf expression. It is also unlikely that Mitf expression is dependent upon extracellular factors (that is, RPE-promoting signals) not normally present in the wild-type retina because Mitf was expressed in *Vsx2*^*orJ*^ RPCs surrounded by wild-type (*Yfp*^+^) retinal cells.

### Regulation of RPC proliferation by Vsx2 is likely to involve both cell-nonautonomous and autonomous mechanisms

That *Vsx2*^*orJ*^ cells respond differently in peripheral versus central regions of mutant chimeras underscores the complexity of how Vsx2 influences proliferation. While previous genetic and molecular studies implicated the intrinsic factors Mitf, p27^Kip1^, and Cyclin D1 in Vsx2-mediated regulation of RPC proliferation [33,77,1,36,4], we demonstrate here that cell-nonautonomous mechanisms involving extrinsic signals also contribute. In *peripheral* regions, the sixfold increase in EdU labeling of *Vsx2*^*orJ*^ cells in mutant chimeras represents at least a partial rescue of proliferation in response to the presence of wild-type *Yfp*^+^ cells. Notably, this rescue occurs despite persistent Mitf expression in *Vsx2*^*orJ*^ cells. In a variety of contexts, Mitf inhibits proliferation through transcriptional activation of several cell cycle inhibitors, including p27^Kip1^ [85-87]; and genetic removal of Mitf in Vsx2-deficient retinas improves retinal size and RPC proliferation [4,1,77]. Since proliferation was not restored to wild-type levels in the peripheral *Vsx2*^*orJ*^ cells in the mutant chimera, it is possible that Mitf still inhibited proliferation. But, the enhanced proliferation reveals the proliferative competence of Vsx2-deficient RPCs when they are exposed to a growth-promoting environment. It remains unclear, however, whether this enhanced proliferation in mutant chimeras reflects restoration of a disrupted retinal mitogen or alleviation of an aberrant anti-proliferative signal. Vsx2-dependent alterations in key developmental extracellular signaling pathways, including those with mitogenic and anti-proliferative activity have been reported [58,88].

Our proliferation analysis in mutant chimeras also revealed significant changes in *central* regions. But here, the presence of wild-type *Yfp*^+^ cells produced the opposite effect, a reduction in EdU labeling of *Vsx2*^*orJ*^ cells. We previously reported reduced hedgehog (Hh) signaling activity in the *orJ* retina, which correlated, in part, with delayed production of RGCs, the primary source of sonic hedgehog (SHH) ligand, an important retinal mitogen [58]. Therefore, we predicted that RGC production from wild-type *Yfp*^+^ cells in retinas of mutant chimeras would restore endogenous SHH and either maintain or increase proliferation of *Vsx2*^*orJ*^ cells, particularly in the central region, where RGC production was active. Our observation of *reduced* proliferation suggests that the endogenous SHH provided by *Yfp*^+^ RGCs was not sufficient to support proliferation of *Vsx2*^*orJ*^ RPCs.

The basis for this reduced proliferation is not clear. It is possible that a mitogen signal unique to the central *orJ* retina was lost in the mutant chimeras or an inhibitory signal normally present in the wild-type retina is lacking in the *orJ* retina. Both explanations seem unlikely given the similar levels of proliferation in central *orJ* and wild-type embryonic retinas (Figure 4E(*c*)) [56,29,30]. Rather, the novel, reduced proliferative activity of central *Vsx2*^*orJ*^ cells in mutant chimeras suggests a cell competition scenario in which *cell-autonomous* differences in cellular fitness are revealed as a result of intercellular interactions between these populations [89]. Here, however, the less-fit cells (*Vsx2*^*orJ*^ RPCs in the central retina) were not eliminated from the tissue compartment. Instead, wild-type cells may have been more efficient at utilizing the available, but likely limited, mitogen signals, at the level of, or downstream of, ligand:receptor interactions. That Vsx2 controls RPC proliferation in part through a cell-autonomous mechanism is supported by our observations that proliferation of *Vsx2*^*orJ*^ cells did not reach wild-type levels in peripheral regions and was unaffected by environmental factors in intermediate regions in mutant chimeras, and by our previous finding that proliferation was acutely enhanced in dissociated *Vsx2*^*orJ*^ cells transfected with *Vsx2* [4].

How can the contrasting responses of the *Vsx2*^*orJ*^ cells in peripheral versus central regions of mutant chimeras be reconciled? First, it is likely that the cell-autonomous role for Vsx2 underlying the observed cell-nonautonomous competition in central regions is active in all RPCs. Second, the finding that a positive growth environment is sufficient to negate the effects of this competition in peripheral and intermediate regions further supports the idea that in mutant chimeras, central wild-type and *Vsx2*^*orJ*^ cells compete for limiting mitogen signals.

### Vsx2 controls the timing of onset of neurogenesis in a cell-autonomous manner

The cell-autonomous delay in neurogenesis exhibited by *Vsx2*^*orJ*^ cells in mutant chimeras was consistent with the delayed neurogenic program observed in the germline mutant retina. The failure of *Vsx2*^*orJ*^ cells to differentiate, despite active neurogenesis in neighboring wild-type *Yfp*^*+*^ cells, demonstrates the inherent, although temporary, inability of *Vsx2*^*orJ*^ cells to respond to neurogenic signal(s). Thus, the delayed onset of neurogenesis in *orJ* retinas results from impaired neurogenic competence as opposed to altered environmental signals.

Intriguingly, *Vsx2*^*orJ*^ cells in mutant chimeras maintained the central-to-peripheral wave of neurogenesis, despite its delayed onset. Thus, two independent, heterochronic waves of neurogenesis were seen in the mutant chimeras: first, the normal central-to-peripheral wave of neurogenesis in wild-type *Yfp*^*+*^ cells, followed by a second central-to-peripheral wave of neurogenesis in *Vsx2*^*orJ*^ cells. According to the sequential induction model, the central-to-peripheral wave of neurogenesis results from signaling by nascent retinal neurons that induces neighboring RPCs to differentiate. Consistent with this, both Hh and FGF signals can induce premature retinal neurogenesis and influence progression of the neurogenic wave in fish and chick [90-92]. However, growing evidence has begun to challenge this model. Peripheral RPCs differentiated despite early physical separation from the central retina in chick [92], and RGC differentiation occurred even when naïve RPCs were transplanted into non-retinal regions of the zebrafish embryo [93]. We found that *Vsx2*^*orJ*^ cells at different central-to-peripheral retinal positions within mutant chimeras do not gain competence all at once, as would be expected for the sequential induction model involving a signal from wild-type cells that had already progressed to the peripheral retina. Furthermore, mosaic conditional inactivation of *Shp2*, an important FGF pathway component, demonstrated the ability of more peripheral wild-type RPCs to differentiate beyond an undifferentiated patch of mutant RPCs [83], suggesting that RPCs do not require direct contact with nascent neurons to initiate neurogenesis. An alternative model argues for cell-autonomous control of the initiation of neurogenesis, suggesting that RPCs differentiate based on a preprogrammed, intrinsic timer. The underlying source of this cell-autonomous ‘clock’ has remained elusive. The proliferative defect in the *orJ* retina could support a model where this clock was tied to cell divisions; however, the changes in proliferation observed in the mutant chimeras had no effect on the timing of neurogenesis onset. The presence of a second central-to-peripheral wave of neurogenesis in *Vsx2*^*orJ*^ cells of chimeric retinas also suggests that there is a strong cell-autonomous component driving the neurogenic wave across the retina. This is consistent with studies in which transplanted zebrafish RPCs expressed *ath5* (RGC determinant) according to their original retinal position, independent of the location into which they were transplanted [93].

### Nonautonomous effects on wild-type RPCs support a model of homeostatic control of fated precursor production

Our investigation of neurogenesis revealed an unexpected change in the cell type distribution of wild-type *Yfp*^*+*^ cells in mutant chimeras at E15.5. In regions with high *Vsx2*^*orJ*^ contribution, *Yfp*^*+*^ cells were largely present as postmitotic precursors, notably RGCs (POU4F+) and cone precursors (OTX+). The lack of the apoptosis marker Casp3 suggests that the absence of *Yfp*^*+*^ RPCs was not due to cell death. *Yfp*^*+*^ and *Vsx2*^*orJ*^ RPCs may have segregated due to differential affinity, and then, through tangential migration, nascent wild-type RGC and cone precursors moved back into *Vsx2*^*orJ*^ regions. However, we find this complex scenario unlikely for several reasons. First, differential affinity between mutant and wild-type cells in Pax6 chimeras resulted in extrusion of the mutant cells into the subretinal space [44,45], a behavior not observed in our chimeras. Second, RGC and cone precursors originating from wild-type RPCs, as indicated by EYFP expression, were found in their normal laminar positions and in direct contact with mutant cells in mutant chimeras. Additionally, there were many examples of interspersed RPCs of mutant and wild-type origin. Lastly, although tangential migration of RGC precursors has been reported as early as E15.5, most migration occurs later, and cone precursors do not undergo tangential migration until postnatal ages [94].

The simplest explanation for the absence of wild-type *Yfp*^*+*^ RPCs within *Vsx2*^*orJ*^ patches is that they underwent neurogenesis at the expense of maintaining themselves as progenitors. Depletion of early embryonic RPCs through precocious neurogenesis initiated by gene disruption or manipulation of signaling can result in the overproduction of earlier born cell type(s) at the expense of later born cell types [54,75,95-100]. We discovered a specific overrepresentation of RGC precursors within the *Yfp*^*+*^ cell population, and this is consistent with a depletion of *Yfp*^*+*^ RPCs into the earliest born cell type (RGCs) that precluded an overrepresentation of the later born cell types (cones, amacrine cells, and horizontal cells).

What could underlie this nonautonomous effect on these cells? Two non-mutually exclusive possibilities are that *Vsx2*^*orJ*^ RPCs failed to provide a progenitor maintenance signal to *Yfp*^*+*^ RPCs, or that precocious differentiation of *Yfp*^*+*^ cells in *Vsx2*^*orJ*^ environments was a secondary effect of the cell-autonomous delay in neurogenesis of *Vsx2*^*orJ*^ cells. Importantly, neither explanation requires that precursors be overproduced at the tissue level. Absence of differentiating cells of *Vsx2*^*orJ*^ origin early in mutant chimeras could have resulted in non-limiting neurogenic signal(s) that drove continued differentiation of *Yfp*^*+*^ cells because *Vsx2*^*orJ*^ cells were incompetent to respond to these signals. Alternatively, reduced neuron production early may have resulted in reduced negative feedback and precocious differentiation of competent cells, in this case, *Yfp*^*+*^ RPCs. Along these lines, differentiated cells have been shown to produce signals that inhibit neurogenesis in adjacent RPCs, such as SHH [90] and VEGF [101].

The scenario that *Yfp*^*+*^ RPCs underwent neurogenesis at their own expense because neighboring *Vsx2*^*orJ*^ RPCs were unable to suggests a strong homeostatic drive to preserve neurogenesis. Since the *Yfp*^*+*^ and *Vsx2*^*orJ*^ RPCs are not of the same genetic origin, this is likely to have occurred through a community effect; *Yfp*^*+*^ RPCs altered their behavior to compensate for the neurogenic deficiency of *Vsx2*^*orJ*^ RPCs to attain the overall goal of balanced cell production. It would be interesting to determine if community effect-like mechanisms are active in the normal developing retina. By transcending clonal boundaries, they would provide environmental input into the seemingly stochastic mechanisms governing progenitor-based decisions (that is, to remain a progenitor or not; precursor fate choice) [102].

## Conclusions

In sum, through the use of genetic chimeras, we have determined the autonomy characteristics of Vsx2 function in embryonic RPCs in the early stages of retinal histogenesis. In addition to the cell-autonomous circuit controlling retinal identity, we found that Vsx2 is upstream of cell-autonomous and cell-nonautonomous mechanisms controlling proliferation, and upstream of a strictly cell-autonomous mechanism to control the timing of onset of retinal neurogenesis (Figure 11). These latter findings underscore a previously unappreciated role for Vsx2 in establishing the extrinsic signals that regulate these important RPC properties. Furthermore, the use of genetic chimeras enabled the disentanglement of several Vsx2 functions. We demonstrated that Vsx2 plays a significant role in proliferation that is independent of its role in promoting retinal identity through repression of Mitf. We also show that the delayed neurogenesis of Vsx2-deficient RPCs is not a secondary effect of their impaired proliferative activity. Future studies can now be directed at identifying the downstream targets and pathways of Vsx2 that control the timing and execution of these essential RPC properties.

## Methods

### Mice

*orJ* mice on a 129S1/Sv background and *Gt(Rosa)26Sor*^*tm1Sor*^ mice [53] were purchased from The Jackson Laboratory (Bar Harbor, ME, USA). *Lhx2* floxed mice (*Lhx2*^*floxed*^; [50]) were kindly provided by Edwin Monuki (University of California, Irvine, CA, USA). The α*-Cre* transgenic mice [51] were kindly provided by Valerie Wallace (Toronto Western Research Institute, Toronto, Ontario, Canada). Tg(CAG-EYFP)7AC5Nagy mice were produced and maintained by the Transgenic and Gene Targeting Mouse Core at the University of Utah (Salt Lake City, UT, USA). Briefly, 7AC5/EYFP ES cells (ATCC, Manassus, VA, USA) were injected into C57BL/6J blastocysts. The 7AC5/EYFP ES cells carry the Tg(CAG-EYFP)7AC5Nagy transgene, in which EYFP is driven by a CMV immediate early enhancer coupled to the chicken β-actin promoter and first intron, on 129X1/S1 background. Chimeric mice were intercrossed to generate homozygotes and the transgene was maintained on (129X1/Svj × 129S1/Sv) × C57BL/6 mixed background. Mice were bred overnight and noon on the day a vaginal plug was observed was considered embryonic day 0.5 (E0.5). All animal use and care was approved by and performed in accordance with the University of Utah Institutional Animal Care and Use Committee, protocol numbers 08-11009 and 11-10010.

### Generation of aggregation chimeras

Chimeric embryos were generated by the Transgenic and Gene Targeting Mouse Core at the University of Utah using morula aggregation techniques (Figure 1). Briefly, eight-cell stage embryos were obtained from three independent homozygous crosses of superovulated females to males of the appropriate strain. The resulting embryos were either homozygous *Vsx2*^*orJ*^, homozygous *Vsx2*^*wt*^, or homozygous Tg(CAG-EYFP)7AC5Nagy (*orJ*, wild-type, or *Yfp* embryos, respectively). Mutant chimeras were generated by aggregating *orJ* embryos with *Yfp* embryos. Control chimeras were generated by aggregating wild-type embryos with *Yfp* embryos. Most chimeras in this study were generated by aggregating two embryos together. However, in order to increase the contribution of *Vsx2*^*orJ*^ cells in the resulting mutant chimeras, some chimeras were generated by aggregation of three embryos (that is, two *orJ* embryos with one *Yfp* embryo). Successfully aggregated chimeric blastocysts were surgically transferred into the uterine horn of E2.5 or oviducts of E0.5 pseudopregnant C57BL/6J × FVB F1 females and allowed to develop to the desired stage. Embryo development was timed according to the pseudopregnancy of the recipient female and staging confirmed according to Theiler [103].

### EdU pulse labeling and detection

Pulse labeling of control and chimeric retinas was performed in retinal explant cultures. Retinas were dissected from surrounding tissues in Hank’s buffered saline solution (HBSS), leaving the lens and vitreal chamber intact. Retinal explants were cultured for 1 h in HBSS containing 33.3 μM 5-ethynyl-2’-deoxyuridine (EdU; Invitrogen-Molecular Probes, Eugene, OR, USA). Cultures were incubated at 37°C and 5% CO_2_, with nutating. Explants were fixed in 4% paraformaldehyde (PFA) in phosphate-buffered saline (PBS, pH 7.5) for 30 min at room temperature, cryoprotected, and stored at −80°C until sectioning. Sections (10 to 12 μm) were cut and stored at −20°C until use. EdU incorporation was detected in cryosections using AlexaFluor568 azide and the Click-iT Cell Reaction (Invitrogen-Molecular Probes, Eugene, OR, USA).

### Immunohistochemistry

Whole eyes or isolated retinas of control and chimeric mice were dissected in HBSS. Whole eyes for use in MITF expression analyses were fixed in 4% PFA in PBS for 2 h at 4°C. Isolated retinas with lenses intact, with or without EdU labeling, were fixed in 4% PFA in PBS for 30 min at room temperature. Following fixation, the tissue samples were cryoprotected and stored at −80°C until sectioning. Sections (10 μm for E12.5 samples and 12 μm for E15.5 samples) were cut and stored at −20°C until staining.

Frozen sections were rehydrated in PBS and pretreated with blocking buffer (2% normal goat or donkey serum, 0.15% TritonX-100, and 0.01% sodium azide in PBS) for 30 min. Primary antibodies are listed in Table 2. Primary antibodies were diluted in the appropriate blocking buffer and incubated overnight at 4°C. Antigen unmasking with 1% sodium dodecyl sulfate (SDS) in PBS was performed prior to blocking pretreatment for the MITF antibody. Primary antibodies were detected using species-specific secondary antibodies conjugated to AlexaFluor568 or 647 (Invitrogen-Molecular Probes, Eugene, OR, USA). In all images, the endogenous EYFP signal was visualized, *without* antibody staining. Nuclei were stained with 4,6-diamidino-2-phenylindole (DAPI; Fluka-Sigma-Aldrich, St. Louis, MO, USA) or TOPRO\u00AE-3 iodide (TOPRO-3; Invitrogen-Molecular Probes, Eugene, OR, USA). Sections were mounted with VECTASHIELD Mounting Medium (Vector Laboratories, Inc., Burlingame, CA, USA).

### Image capture and processing

All immunofluorescence images were captured on an Olympus Fluoview 1000 confocal microscope (Olympus America Inc., Center Valley, PA, USA). Images were prepared for quantification and publication using Photoshop CS5 Extended and Illustrator CS6 (Adobe Systems Inc., San Jose, CA, USA), except where noted. Olympus Fluoview confocal files were first imported using the Bio-Formats Plugin [104] with NIH ImageJ [105] or Fiji [106].

### Marker quantification and analysis

All statistical analyses were performed in Jmp Pro 11.0 (SAS Institute, Inc., Cary, NC, USA).

#### Proliferation

EdU labeling indices were calculated by regional quantification of EdU+ cells in E12.5 retinas. Single-slice confocal images of retinal sections were divided into six bins (central, intermediate, and peripheral in both retinal hemispheres) using ImageJ or Fiji. In retinal sections containing an optic nerve head, retinal hemispheres were divided according to the position of the optic nerve head. Each retinal hemisphere was then further subdivided into three bins (central, intermediate, and peripheral). This was accomplished by first drawing a line from the center of the optic nerve head to the peripheral tip of the retina, which splits the retina’s width at the apical-basal midpoint. This line was then divided into three equal segments and a perpendicular line extended to both apical and basal edges. In retinal sections lacking an obvious optic nerve head, the line drawn at the apical-basal midpoint was drawn from one peripheral tip to the other and divided into six equal segments. Central sections taken from at least three animals were analyzed per condition. For each section, the number of EdU-labeled and total EYFP+ or EYFP− cells were counted for central, intermediate, and peripheral bins of chimera, *orJ*, and wild-type retinas. In sections of mutant chimeras with very low *Vsx2*^*orJ*^ contribution, only *Yfp*^*+*^ cells within an apical-basal column located within two cell diameters of a patch of *Vsx2*^*orJ*^ cells or regions with exposure to *Vsx2*^*orJ*^ cells were counted. Counts were summed within regions across sections from the same animal. The proliferating population was calculated as a percentage of the total EYFP+ or EYFP− population and compared within corresponding regions across *orJ*, wild-type, and chimeric retinas. Statistically significant differences between *Vsx2*^*wt*^ and *Vsx2*^*orJ*^ populations in *orJ*, wild type, and chimeras were determined by Tukey-Kramer HSD. For comparison of *Yfp*^*+*^ populations in mutant and control chimeras, Student’s unpaired *t*-test or Welch’s two-sample *t*-test, as appropriate (based on results of an F-test for equal variance), was used.

#### Neurogenesis

##### Contribution of Vsx2^orJ^ and Vsx2^wt^ cells to the RGC precursor pool in chimeric retinas at E12.5

*Vsx2*^*orJ*^ and *Vsx2*^*wt*^ cells were identified by their lack of EYFP expression (EYFP−) in mutant and control chimeras, respectively. Cell counts were performed on single-slice confocal images in Photoshop CS5 Extended. For each section, the full complement of POU4F+ cells and the cohort of POU4F+ EYFP− cells were counted. The contribution of POU4F+ EYFP− cells was calculated as a percentage of the total POU4F+ cells for each chimera type.

Since the cell contribution from each strain could be influenced by the extent of chimerism, we estimated the contribution of EYFP− cells to the retina on each section in the regions where POU4F+ cells were counted defined here as the *region of interest* (ROI). To do this, we calculated the percentage of pixels that was assigned to a bin designated as *EYFP*− in each ROI using ImageJ. Briefly, ROIs were set for each image by outlining the entire apical-basal extent of retina in the regions where POU4F+ cells were found using *ROI Manager*. The ROI was loaded into a single channel gray scale image (EYFP) for each sample; the default *Smooth* function was applied, followed by manual *Brightness* adjustment. Manual *Threshold* was applied, and the cutoff was based on a visual evaluation of the correspondence of the mask with the perceived EYFP fluorescence pattern. All pixels in the ROI had a gray scale value of 0 (EYFP+) or 255 (EYFP−). The number of pixels corresponding to the EYFP− bin and the total number of pixels were calculated using the *Histogram* function. The contribution of EYFP− cells to the chimera was calculated as a percentage of the total number of pixels in the ROI. This value was then linked to the POU4F cell count value for each sample and graphed as a bivariate plot. Linear regression plots were generated from a standard least squares fit with an interaction term to allow for unequal slopes. This was followed by an F-test for analysis of covariance to determine whether the difference in the slopes of the regression lines was statistically significant.

##### Contribution of Yfp^+^ cells to RGC and cone precursor production in mutant chimeras at E15.5

The relative ratios of POU4F+ and OTX+ *Yfp*^*+*^ cells in *Vsx2*^*orJ*^ versus *Yfp*^*+*^ cell patches were calculated. *Yfp*^*+*^ and *Vsx2*^*orJ*^ cell patches in single-slice confocal images were manually masked in Photoshop CS5 Extended. Regions of high *Vsx2*^*orJ*^ contribution were classified as *Vsx2*^*orJ*^ patches and defined by extending perpendiculars on either side at the outermost contiguous *Vsx2*^*orJ*^ cell. Regions of *Vsx2*^*orJ*^ cells were split into separate *Vsx2*^*orJ*^ patches if 3 or more *Yfp*^*+*^ cell widths spanned the retina to divide adjacent groups of *Vsx2*^*orJ*^ cells. *Yfp*^*+*^ patches contained few or no *Vsx2*^*orJ*^ cells and were defined by extending perpendiculars on either side at least three cell widths from the nearest *Vsx2*^*orJ*^ patch. Differentiated *Yfp*^*+*^ cells (marker+, EYFP+) and total *Yfp*^*+*^ cells (EYFP+) were counted in all masked patches of mutant chimeras. Differentiation of the *Yfp*^*+*^ population was calculated as a percentage of the total *Yfp*^*+*^ cells and compared across patch type. Statistical significance was determined using Student’s unpaired *t*-test or Welch’s two-sample *t*-test, as appropriate (based on results of an F-test for equal variance).

## Additional files

Additional file 1:
**Anti**-**mouse immunoreactivity control. (A)** Nonspecific staining due to the anti-mouse secondary antibody along the vitreal edges of the lens and retina, in the developing corneal epithelium and extraocular mesenchyme, but not in the RPE or retina of a wild-type eye under the MITF immunostaining conditions. Dashed lines demarcate the neural retina from the RPE. Scale bars: 100 μm (left panel); 40 μm (right panel). Abbreviations: NR, neural retina; RPE, retinal pigmented epithelium. Additional file 2:
***Vsx2*** ^***WT***^ **cells contribute to all early born retinal cell types in E15.5 control chimeras.** Expression of TUBB3 **(A)**, POU4F **(B)**, OTX **(C)**, PTF1A **(D)**, and BHLHB5 **(E)** in retinas of control chimeras at E15.5. Scale bars: 100 μm. Additional file 3:
**Low levels of cell death are detected in non-chimeric and chimeric retinas.** Expression of activated caspase-3 (CASP3) in retinas of wild type, *orJ*, control chimeras, and mutant chimeras at E12.5 **(A-F)**, E14.5 **(G)**, and E15.5 **(H-K)**. Insets and arrows show retinal cells stained for CASP3. Scale bars: 100 μm. Note size difference in scale bars (A, G, H, I, J, K).

## Abbreviations

β-gal: β-galactosidase
E: embryonic day
EdU: 5-ethynyl-2′-deoxyuridine
EYFP: enhanced yellow fluorescent protein
Hh: hedgehog
*or*: *ocular retardation*
*orJ*: *ocular retardation J*
RGC: retinal ganglion cell
RPC: retinal progenitor cell
RPE: retinal pigment epithelium
Shh: sonic hedgehog
