# Supplementary figures and images for: Genetic chimeras reveal the autonomy requirements for Vsx2 in embryonic retinal progenitor cells
Source: Neural Dev. 2015 Apr 27;10:12. doi: 10.1186/s13064-015-0039-5 (PMC4450477; doi:10.1186/s13064-015-0039-5)

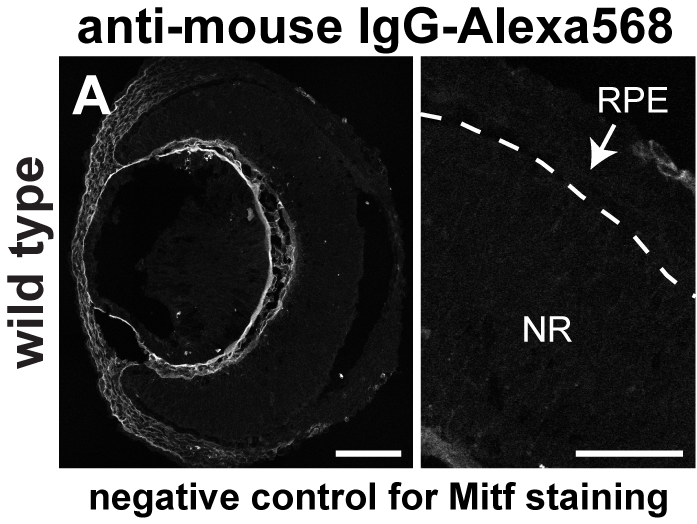

Supplement: Additional file 1: — Anti-mouse immunoreactivity control. (A) Nonspecific staining due to the anti-mouse secondary antibody along the vitreal edges of the lens and retina, in the developing corneal epithelium and extraocular mesenchyme, but not in the RPE or retina of a wild-type eye under the MITF immunostaining conditions. Dashed lines demarcate the neural retina from the RPE. Scale bars: 100 μm (left panel); 40 μm (right panel). Abbreviations: NR, neural retina; RPE, retinal pigmented epithelium. [file 13064_2015_39_MOESM1_ESM.tiff]

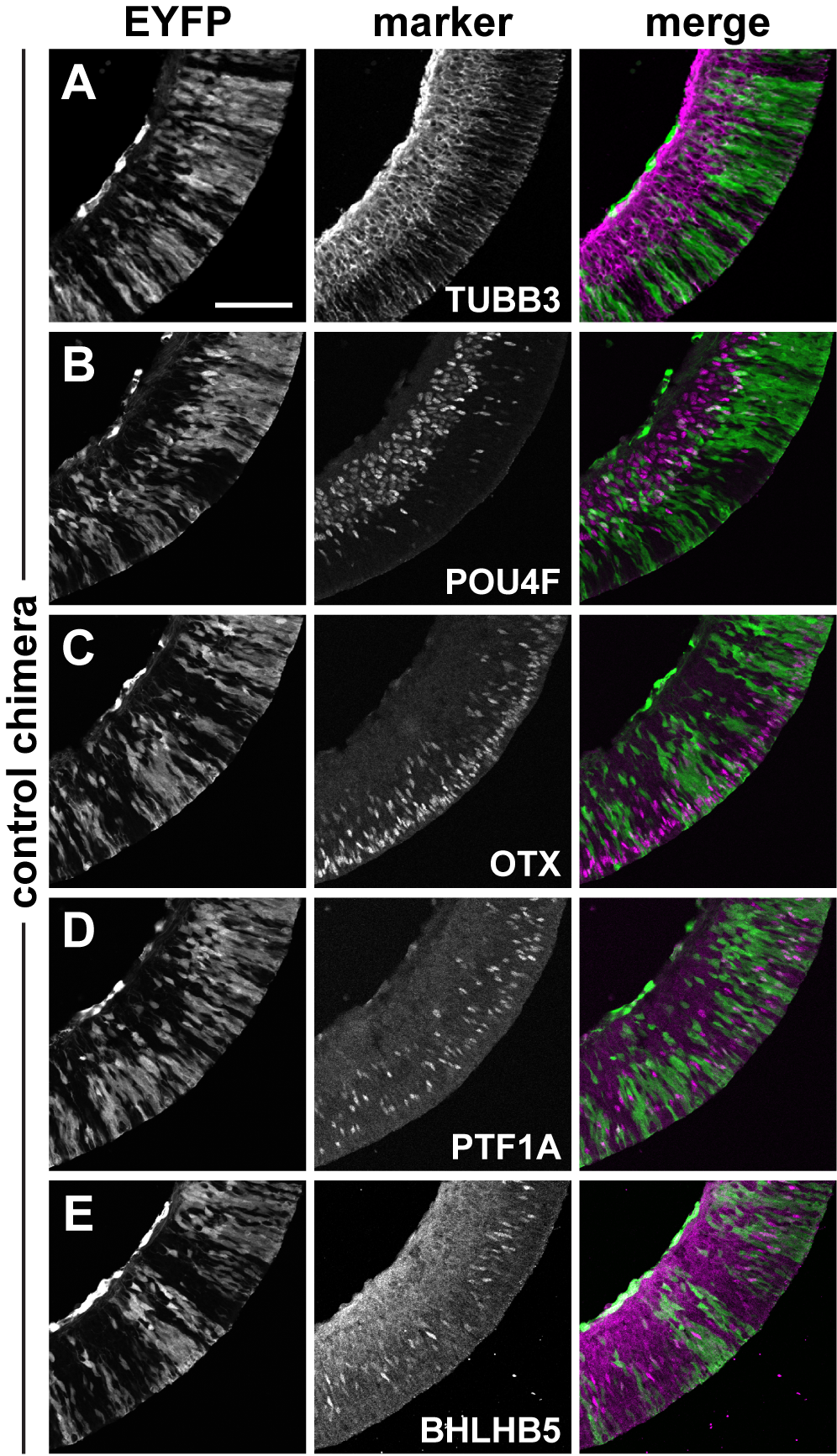

Supplement: Additional file 2: — Vsx2 WT cells contribute to all early born retinal cell types in E15.5 control chimeras. Expression of TUBB3 (A), POU4F (B), OTX (C), PTF1A (D), and BHLHB5 (E) in retinas of control chimeras at E15.5. Scale bars: 100 μm. [file 13064_2015_39_MOESM2_ESM.tiff]

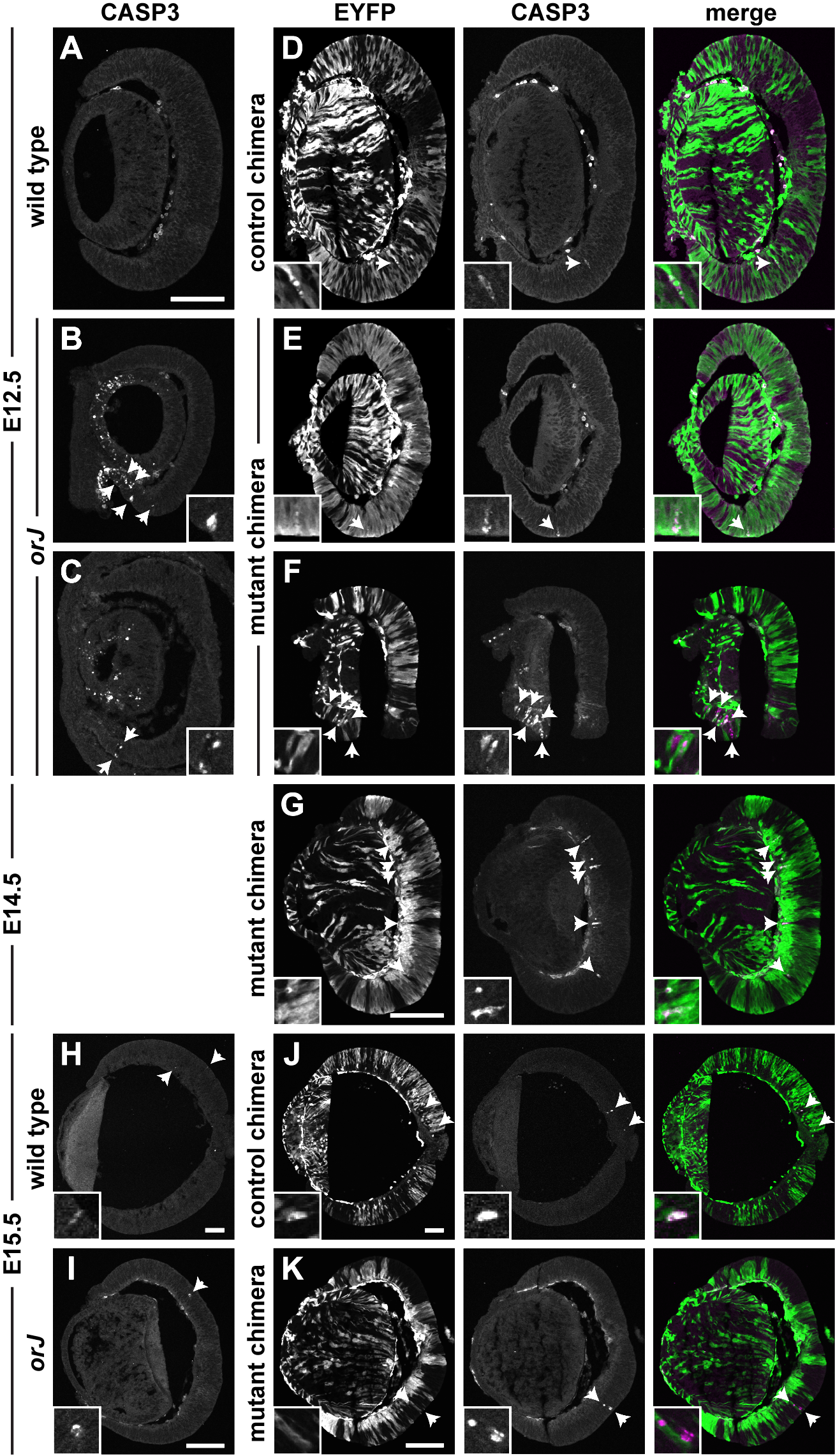

Supplement: Additional file 3: — Low levels of cell death are detected in non-chimeric and chimeric retinas. Expression of activated caspase-3 (CASP3) in retinas of wild type, orJ, control chimeras, and mutant chimeras at E12.5 (A-F), E14.5 (G), and E15.5 (H-K). Insets and arrows show retinal cells stained for CASP3. Scale bars: 100 μm. Note size difference in scale bars (A, G, H, I, J, K). [file 13064_2015_39_MOESM3_ESM.tiff]
